# TIGIT Expression and Its Implications in Non-Small-Cell Lung Cancer Progression and Therapy: A Systematic Review

**DOI:** 10.3390/ijms26199307

**Published:** 2025-09-23

**Authors:** Julia Piekarz, Natalia Picheta, Katarzyna Szklener, Sławomir Mańdziuk

**Affiliations:** 1 Student Academic Group, Department of Clinical Oncology and Chemotherapy, Medical University, 20-090 Lublin, Poland; piekarzjulia1@gmail.com (J.P.);; 2 Department of Clinical Oncology and Chemotherapy, Medical University, 20-090 Lublin, Poland

**Keywords:** Non-Small-Cell Lung Cancer, TIGIT, TIGIT inhibitors, tiragolumab

## Abstract

Lung cancer (LC) is the leading cause of cancer-related mortality worldwide, with non-small-cell lung cancer (NSCLC) representing 85–90% of cases. Despite the efficacy of PD-1/PD-L1 immune checkpoint inhibitors, primary and acquired resistance highlight the need for novel immunotherapeutic strategies. A systematic review of the literature from 2020 to 2025 was conducted according to the PICO model. Six studies were included, encompassing phase I–III clinical trials. The analysis focused on efficacy, safety, and emerging therapeutic strategies targeting TIGIT in NSCLC. TIGIT blockade enhances cytotoxic T lymphocyte and natural killer (NK) cell activity, strengthening antitumor immunity. Clinical trials, particularly with the monoclonal antibody tiragolumab combined with PD-1/PD-L1 inhibitors, show promising synergistic effects. Emerging strategies, including bispecific antibodies (e.g., TIGIT/PD-1 and TIGIT/PD-L1) and experimental cell therapies, are under investigation to further improve the antitumor response. Anti-TIGIT therapies represent a highly promising approach in NSCLC. While phase III data remain limited, biomarker-driven, well-designed trials are essential. If validated, TIGIT blockade could become a key addition to immuno-oncology treatment strategies for NSCLC.

## 1. Introduction

Lung cancer (LC) remains one of the most common malignant tumors and the leading cause of cancer deaths worldwide, accounting for over 25% of all cancer deaths. Approximately 2 million new cases are diagnosed annually worldwide, of which approximately 1.76 million result in patient death. Most deaths are related to smoking, and mortality rates vary by gender, with fewer cases reported in women [1]. The most important risk factor, occurring in about 85% of cases, is smoking. Additional risk factors include exposure to fine particulate matter, chemicals such as asbestos or radon [2]. Genetic factors, family history, and certain viral infections such as Human Papillomavirus (HPV) and bacterial infections (e.g., *Mycobacterium tuberculosis*) also significantly increase the risk of developing LC [3].

Histologically, approximately 15% of cases are small-cell lung cancer (SCLC), while 85% are non-small-cell lung cancer (NSCLC). The main subtypes of NSCLC include adenocarcinoma, squamous cell carcinoma, and large cell carcinoma, of which adenocarcinoma is the most common [4]. In addition, NSCLC is characterized by a diverse molecular profile, in which activating mutations in genes such as epidermal growth factor receptor (EGFR), Kirsten rat sarcoma viral oncogene homolog (KRAS), and anaplastic lymphoma kinase (ALK) rearrangements play a key role. Less commonly observed are molecular changes in the mesenchymal–epithelial transition factor (MET), human epidermal growth factor receptor 2 (HER2), c-ros oncogene 1 (ROS1), B-Raf proto-oncogene (BRAF) or rearranged during transfection (RET) genes, as well as changes in suppressor genes such as tumor protein p53 (TP53). The presence of these mutations determines the choice of targeted therapy, making molecular profiling an integral part of modern NSCLC treatment [5].

An important element of NSCLC is its microenvironment, which plays a key role in the immune response. The expression of immune checkpoints receptors such as Programmed Death-Ligand 1 (PD-L1) and T-cell immunoreceptor with Ig and ITIM domains (TIGIT) modulates the activity of T lymphocytes and Natural Killer cells (NK), and thus the effectiveness of immunotherapy. Therapeutic agents targeting these receptors, rather than the receptors themselves, can be blocked, especially in combination therapy, and represent a promising strategy for increasing T-cell activity and improving treatment outcomes [6]. Due to the high incidence of NSCLC, its aggressive course, and persistently high mortality rate, new therapeutic strategies are being intensively sought, both as a supplement to current treatment methods and as potential standalone therapeutic options [6].

NSCLC therapy is based on surgery, radiotherapy, chemotherapy, immunotherapy, and molecularly targeted therapy. Each of these methods can be used separately or in combination. The choice of therapeutic method depends on the stage of the disease, histology, patient condition, and genetic changes [7]. It has been found that PD-L1-based immunotherapy can significantly prolong survival from 5% to as much as 29.6%. However, the increasing incidence of primary and secondary resistance significantly indicates the need to search for other immunotherapy-based methods [8]. Despite advances in the treatment of NSCLC, high mortality and limited efficacy of some immunotherapies, especially in cases of primary or secondary resistance to Programmed Death-1 (PD-1)/PD-L1 inhibitors, remain a challenge. In this context, there is growing interest in the TIGIT receptor, an immune checkpoint receptor for T and NK cells, whose expression in the tumor microenvironment may inhibit the immune response. Preclinical studies and early clinical trials indicate that simultaneous blockade of PD-1/PD-L1 and TIGIT may synergistically increase cytotoxic cell activity and improve treatment efficacy, opening up new prospects for the treatment of NSCLC [7].

Unlike previous reviews on TIGIT in NSCLC, this systematic review provides a distinct perspective by focusing primarily on the most recent studies published between 2022 and 2025, including the latest translational analyses and early- to late-phase clinical trials. In particular, we integrate detailed insights into the molecular mechanisms of the TIGIT/CD226 axis with up-to-date clinical trial outcomes (phase I–III). We place special emphasis on the discrepancies observed between early-phase (CITYSCAPE) and late-phase (SKYSCRAPER-01) trials, offering potential explanations rooted in biological heterogeneity and patient selection. Furthermore, we highlight innovative therapeutic avenues, including bispecific antibodies and cellular therapies targeting TIGIT, which extend beyond conventional PD-1/PD-L1–based strategies. Finally, we underscore the urgent need for reliable predictive biomarkers—such as PD-L1, CD155, and the TIGIT/CD226 ratio—to improve patient stratification and optimize the clinical utility of anti-TIGIT therapies. Taken together, this comprehensive, up-to-date mechanistic–translational–clinical approach distinguishes our systematic review from prior publications and provides a forward-looking perspective for the development of TIGIT-targeted strategies in NSCLC.

## 2. Materials and Methods

### 2.1. Review Design and Search Strategy

The research focused on comparing these therapies with conventional treatments. The PICO model was used to organize and guide the literature review.

- Population: The primary population included patients diagnosed with NSCLC at any stage who received TIGIT-targeted therapy, either alone or in combination with other immunotherapies.
- Intervention: Interventions of interest included monoclonal antibodies targeting TIGIT (e.g., tiragolumab, vibostolimab) administered as monotherapy or in combination with PD-1/PD-L1 inhibitors. These therapies were selected based on their mechanism of action in modulating immune checkpoints to enhance antitumor immune responses.
- Comparison: Comparative analysis included standard-of-care treatments (e.g., PD-1/PD-L1 inhibitors alone, chemotherapy) or placebo, depending on the study design.
- Outcome: Outcomes analyzed focused on efficacy (objective response rate [ORR], progression-free survival [PFS], overall survival [OS], duration of response 450544 [DOR]) and safety (treatment-related adverse events [TRAEs], tolerability). Additionally, studies analyzing PD-L1 and TIGIT expression were included to evaluate prognostic and predictive biomarker potential.

The inclusion criteria were original studies conducted in humans with NSCLC, including randomized controlled trials (RCTs) of phase II–III, phase I clinical trials, and retrospective observational studies, evaluating TIGIT-targeted therapies either as monotherapy or in combination with PD-1/PD-L1 inhibitors or chemotherapy, and published in English between 2022 and 2025. Studies were excluded if they were preclinical (in vitro or animal models), case reports, conference abstracts, editorials, letters, focused on cancers other than NSCLC, or were not available in full text.

The search strategy covered publications from 2022 to 2025 in PubMed, Scopus and ClinicalTrials.gov. Keywords used included “TIGIT,” “non-small cell lung cancer,” “TIGIT inhibitors,” and “tiragolumab”. Manual screening of retrieved studies ensured inclusion of all relevant trials. Two researchers independently performed the search and critically assessed the selected articles, and any discrepancies were resolved through discussion with a third reviewer.

### 2.2. Collecting Data

The review included 6 studies of X patients, comprising 3 early-phase clinical trials (phase I), 2 RCTs (phase II–III), and 1 retrospective observational study analyzing PD-L1 and TIGIT expression. Data were systematically tabulated to facilitate comparative analysis of outcomes across studies. Data extracted from the included studies encompassed patient characteristics, PD-L1 expression levels, intervention details, study design, and reported outcomes. Efficacy was analyzed through ORR, PFS or and OS, while safety data included TRAEs and tolerability.

### 2.3. Selection and Identifications of Studies

**Figure 1:**
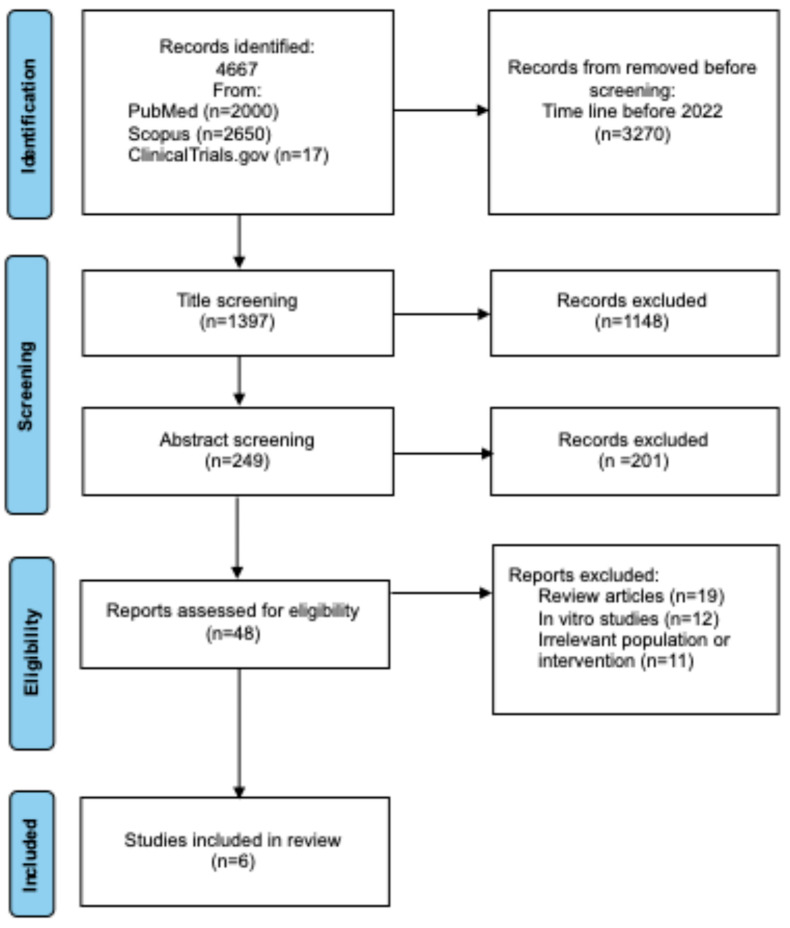
Preferred Reporting Items for Systematic Reviews and Meta-analyses (PRISMA) flow diagram of study identification, inclusion, and exclusion.

A systematic database search identified 4667 records. Of these, 3270 were excluded for being published before 2022. The remaining 1397 records underwent title screening, during which 1148 were excluded as irrelevant. Subsequently, 249 abstracts were reviewed, and 201 were excluded. The full texts of 48 articles were then assessed for eligibility. Following detailed evaluation, 42 studies were excluded for not meeting the inclusion criteria. Ultimately, 6 studies fulfilled all criteria and were incorporated into the final analysis. The comprehensive data selection and identification process is illustrated in Figure 1.

### 2.4. Assessment of Risk of Bias in the Included Studies

The two randomized controlled trials were evaluated using the Cochrane Risk of Bias tool for randomized trials (RoB 2.0) across five domains: bias arising from the randomization process, deviations from intended interventions, missing outcome data, outcome measurement, and selection of reported results. Both trials were assessed independently by two reviewers, and any discrepancies were resolved through discussion with a third reviewer. The three early-phase clinical trials were assessed descriptively for methodological rigor, including sample size, study design, and completeness of outcome reporting. The retrospective observational study was evaluated using the ROBINS-I tool for non-randomized studies, focusing on potential confounding, selection bias, measurement of interventions, and outcome assessment. This structured approach ensured a comprehensive evaluation of methodological quality and potential sources of bias, thereby enhancing the reliability of the systematic review’s findings.

## 3. TIGIT Expression in Non-Small-Cell Lung Cancer

TIGIT is classified as an immune checkpoint inhibitor (ICI). By blocking the activity of T and NK cells, TIGIT limits excessive immune response while inhibiting the response against cancer cells. Blocking TIGIT restores the normal induction, activation, and expansion of tumor-specific cytotoxic T cells, resulting in a sustained response to cancer therapy. In recent years, the US Food and Drug Administration (FDA) has approved several ICIs, including PD-1, cytotoxic T lymphocyte antigen-4 (CTLA-4), and lymphocyte activation gene-3 (LAG-3). ICIs are currently the foundation of immunotherapy for many solid tumors, including NSCLC, and TIGIT is emerging as a potential new therapeutic target [9].

TIGIT is a protein composed of an extracellular immunoglobulin (Ig) variable domain, a type 1 transmembrane domain, and a cytoplasmic tail that has two inhibitory motifs, including an immunoreceptor tyrosine-based inhibitory motif (ITIM) and an Ig tail-tyrosine (ITT)-like motif [10]. This protein is also known as Washington University cell adhesion molecule (WUCAM), V-set and transmembrane domain-containing protein 3 (Vstm3) and V-set and immunoglobulin domain-containing protein 9 (VSIG9) [11].

TIGIT expression has been found in many solid tumors such as melanoma, colorectal cancer, and NSCLC [12]. Currently, five ligands are attributed to it: CD115, also known as polio virus receptor (PVR), CD112 (Nectin2), CD113 (Nectin3), Nectin4, and Fab2 [11]. Among them, the interaction with CD155, which is widely exposed on the surface of many cancer cells and antigen-presenting cells, is of the greatest biological and clinical significance. High CD155 expression correlates with a more aggressive tumor phenotype, increased cancer cell proliferation, and poorer prognosis. CD112 and CD113, present in epithelial and tumor tissues, among others, also participate in the regulation of the immune response by modulating the activity of T and NK cells. The expression of Nectin-4, previously known primarily for its role in cell adhesion, has been linked to the progression of several types of cancer, including lung cancer, and its role in interaction with TIGIT is the subject of intensive research. An interesting example is the bacterial protein Fap2 from Fusobacterium nucleatum, which can also bind TIGIT, suggesting a potential role for the microbiome in modulating the antitumor response through this pathway [13,14].

Tumor-infiltrating lymphocytes (TILs) play a key role in the antitumor immune response. Immunohistochemical analyses have shown that TIGIT is overexpressed in a significant proportion of TILs in many types of cancer, including NSCLC. Retrospective studies have shown that approximately 40–60% of CD8+ TILs in the tumor microenvironment express TIGIT, which correlates with features of immune exhaustion and poorer clinical prognosis [15].

In the context of NSCLC, the role of TIGIT has been well documented in numerous translational analyses and clinical studies. High expression of TIGIT on CD8+ T lymphocytes and NK cells in the tumor microenvironment has been shown to correlate with the phenomenon of immune exhaustion and a weakened antitumor response. At the same time, overexpression of the main TIGIT ligand, CD155, on lung cancer cells promotes the formation of an immunosuppressive microenvironment and supports mechanisms of tumor escape from immune surveillance [16]. Immunohistochemical studies have shown that 85.8% of squamous cell lung cancer samples expressed PVR, while PD-L1 was found in only 26.8% of cases [17]. Importantly, retrospective studies have shown that NSCLC patients with high expression of TIGIT and CD155 had a poorer prognosis and shorter overall survival. For this reason, TIGIT is considered a potential prognostic biomarker, and blockade of the TIGIT/CD155 pathway is a promising therapeutic strategy, especially in combination with PD-1/PD-L1 inhibitors, as confirmed by early clinical trial results [18].

In summary, TIGIT and its ligands exhibit considerable intratumoral heterogeneity in NSCLC, including across different adenocarcinoma growth patterns. While this variability may influence the local immune microenvironment and the activity of T and NK cells, there is currently no evidence that it translates into clinically significant differences in response between histological subtypes. A more promising approach appears to be the evaluation of molecular and immunological biomarkers, such as PD-L1, CD155, or the TIGIT/CD226 ratio, for precise patient selection in anti-TIGIT therapy. Furthermore, integrating these markers with the assessment of other immune checkpoints may help optimize combination therapies, enhancing treatment efficacy while minimizing TRAEs [19].

## 4. Mechanism of Action of TIGIT

### 4.1. Direct Intracellular Signaling

The cytoplasmic domain of TIGIT contains an inhibitory ITIM and an ITT-like motif, which enables it to transmit inhibitory signals [11]. Most of the knowledge about this pathway comes from studies on NK cells. In humans, the ITIM is key—its mutation abolishes TIGIT’s ability to block NK cytotoxicity. In mice, both ITIM and ITT motifs can independently transmit inhibitory signals, and only their combined mutation eliminates this effect [19]. In human NK cells, phosphorylation of the ITT motif allows the recruitment of adaptor proteins such as growth factor receptor-associated protein 2 (Grb2) and β-arrestin 2. Grb2 attracts Src homology 2 (SH2) inositol-1 (SHIP-1), which inhibits phosphoinositide 3 kinase (PI3K) and mitogen-activated protein kinase (MAPK), limiting NK cytotoxicity, while β-arrestin 2 also recruits SHIP-1, leading to inhibition of NF-κB and interferon gamma (IFN-γ) production. In addition, TIGIT signaling reduces the phosphorylation of extracellular signal-regulated kinases 1/2 (ERK1/2) and zeta-chain-associated protein kinase 70/ spleen tyrosine kinase (ZAP70/Syk), which also weakens NK activity [20].

In T lymphocytes, the inhibition mechanism is more difficult to grasp. It is known that TIGIT acts intracellularly, as its activation suppresses the TCR/CD28 response. A decrease in the expression of genes related to activation and the cell cycle is also observed. However, it has not been possible to clearly identify proteins that bind to the cytoplasmic tail of TIGIT. Although it has a sequence resembling ITIM, the presence of a large phenylalanine at position pY+1 may prevent effective binding of SHP2 phosphatases, such as SHP1 [21]. Furthermore, TIGIT does not contain the ITSM motif responsible for SHP2 recruitment in PD-1, which may explain the lack of interaction between TIGIT and these phosphatases [22].

### 4.2. Regulation of CD226 Activity

CD226 (DNAM-1, Nectin-2) is a costimulatory receptor with a wide range of expression, including not only T lymphocytes and NK cells, but also Natural Killer T cells (NKT), B lymphocytes, monocytes, macrophages, dendritic cells, megakaryocytes, platelets, hematopoietic precursor cells, endothelium, and mast cells [23]. In CD8+ T cells, CD226 plays an important role in initiating the immune response, supports the formation of immune synapses through interaction with PVR on antigen-presenting cells, and as an adhesion molecule facilitates the migration of effector memory lymphocytes to inflammatory tissues and tumors [24].

Due to its higher affinity for PVR and CD112 ligands, TIGIT effectively competes with CD226, blocking its activation and weakening signaling. In addition, TIGIT can interact directly with CD226, destabilizing its homodimers and limiting its activating functions, independently of its cytoplasmic domain. CD226 is also regulated by PD-1, whose complex with SHP2 dephosphorylates CD226, suppressing its activity [25].

Activation of CD226 leads to the phosphorylation and degradation of the transcription factor forkhead box protein O1 (FOXO1), which normally inhibits the effector functions of CD8+ T cells, NK cells, and regulatory T cells. Thus, CD226 enhances effector activity, but it can also limit Treg suppressiveness by affecting forkhead box P3 (FOXP3) and other transcription factors such as T-box expressed in T cells (T-BET), eomesodermin (EOMES), and Transcription factor 7 (TCF7). The competition between TIGIT and CD226 for PVR therefore plays a key role in regulating the balance between the effector and suppressive responses of the immune system [26].

Single-cell RNA-seq analyses have shown that in infiltrating CD8+ T lymphocytes, different cell subsets differ in the expression of costimulatory receptors: effector lymphocytes (T EFF) and tissue-resident lymphocytes (T RM) predominantly express CD226, while central/effector memory lymphocytes (T EM) cells predominantly express CD28. Cells that are double-positive for CD226 and CD28 are characterized by an activated or proliferative profile [22]. This arrangement suggests that CD226 plays a key role in the antitumor response of CD8+ T cells and may even compensate for the lack of CD28 in some populations.

Both CD28 and CD226 appear to be important for the effectiveness of cancer immunotherapy. CD28 determines the response to PD-1 blockade, while CD226 is essential for achieving a therapeutic effect in treatment with anti-PD-1/PD-L1 and anti-TIGIT antibodies. High expression of CD226 in CD8+ T cells correlates with a better clinical prognosis and longer survival in patients undergoing immunocompetent therapies [27].

At the same time, reduced expression of CD226 in CD8+ T cells can lead to their dysfunction. Overexpression of the EOMES transcription factor directly suppresses CD226 gene transcription, and interaction with the PVR ligand can initiate ubiquitination and degradation of CD226 via the E3 ubiquitin–protein ligase (Cbl-b) pathway [24]. These processes lead to receptor internalization and loss of function, which has been observed in both CD8+ T lymphocytes and NK cells.

Understanding the balance within the TIGIT/CD226/PVR axis is therefore crucial for optimizing patient selection and improving the efficacy of anti-TIGIT therapies in NSCLC.

**Figure 2:**
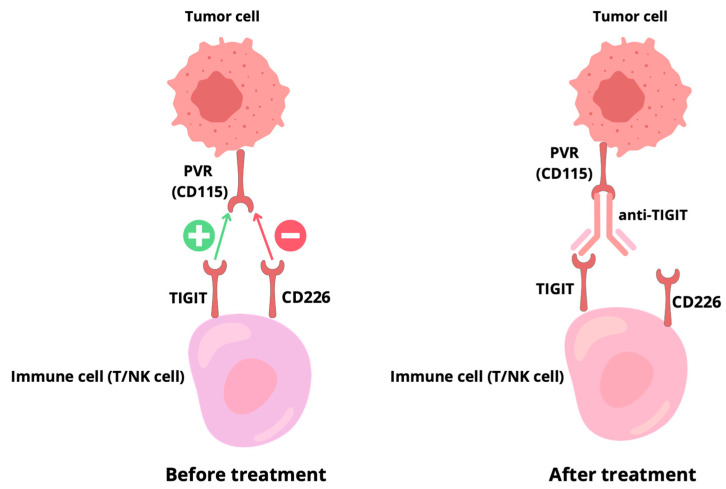
Mechanism of TIGIT blockade. Left: Interaction between tumor cell PVR (CD155) and immune cell TIGIT inhibits activation, competing with the co-stimulatory receptor CD226. Right: Anti-TIGIT antibody blocks TIGIT, allowing CD226-mediated activation and immune response restoration [27].

The TIGIT/CD226/PVR axis and its therapeutic targets are summarized in Figure 2.

### 4.3. Reverse Signaling by PVR Can Modulate the Immune Microenvironment

PVR is constitutively present on epithelial and myeloid cells, and its expression is greatly increased in the tumor environment, particularly in tumor cells and tumor-associated myeloid cells [28]. High PVR levels are associated with poorer clinical response to anti-PD-1 immunotherapy in both metastatic melanoma and non-small-cell lung cancer, and the combined assessment of PVR and PD-L1 may be predictive of treatment efficacy [29].

Unlike PD-L1, whose expression is induced by IFN-γ, PVR is more widespread, also occurring on normal cells. This suggests that it acts as a regulator of the balance between TIGIT-dependent inhibition and CD226-mediated activation. The presence of PVR on dendritic cells is particularly important because, in combination with PD-L1, it may determine patients’ response to checkpoint blockade [30].

The cytoplasmic domain of PVR contains an ITIM, indicating the possibility of reverse signaling. However, its significance is poorly understood. It is known that activation of PVR by TIGIT-Fc in dendritic cells promotes IL-10 secretion and suppression of IL-12, which limits the T-cell response and may support a state of tolerance [31]. Similarly, TIGIT inhibits macrophage activation and shifts their phenotype from pro-inflammatory M1 to immunosuppressive M2. In the tumor microenvironment, MDSCs overexpress both PVR and PD-L1, which further enhances their inhibitory capabilities. Furthermore, PD-L1 blockade increases PVR expression in MDSCs, while TIGIT blockade increases PD-L1 expression, suggesting that only a combined approach can effectively overcome the suppression exerted by these cells [32].

**Figure 3:**
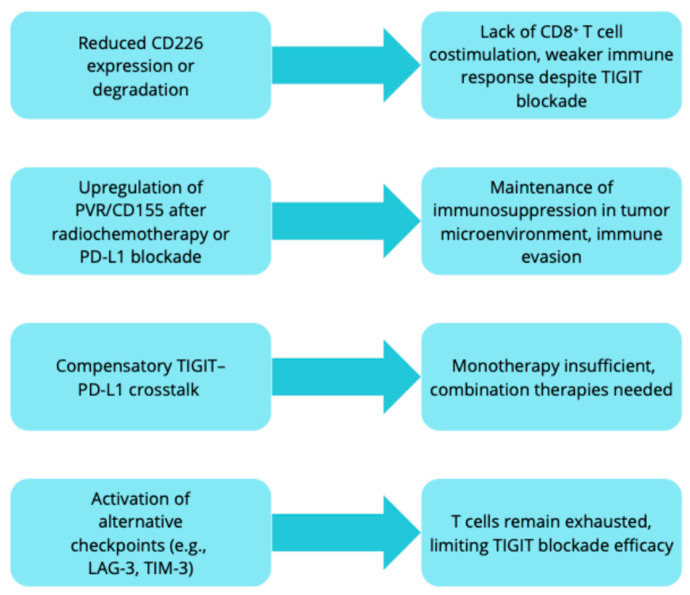
Mechanisms of resistance to TIGIT blockade in NSCLC.

As summarized in Figure 3, multiple mechanisms can limit the efficacy of TIGIT inhibition in NSCLC, highlighting the need for rational combination strategies to overcome resistance.

## 5. TIGIT-Targeted Drugs

The discovery of ICI and the ongoing expansion of this field have revolutionized cancer treatment [33]. TIGIT is one of the most promising targets for NSCLC immunotherapy. This receptor is highly expressed on regulatory T cells and NK cells in the tumor microenvironment, where it plays a key role in suppressing the immune response and facilitating tumor escape from immune surveillance. By interacting with ligands such as CD155, TIGIT inhibits the proliferation and activity of cytotoxic T lymphocytes, promoting the formation of an immunosuppressive tumor microenvironment [34]. TIGIT blockade is therefore a potential anticancer strategy, enabling the restoration of the antitumor response and improving the efficacy of combination therapies, including those with PD-1/PD-L1 inhibitors [35].

In response to the growing interest in immunotherapy in oncology, intensive work has begun on the development of drugs targeting TIGIT blockade. The most advanced therapeutic strategy involves monoclonal antibodies, which bind to the TIGIT receptor on the surface of T lymphocytes and NK cells, thereby abolishing the inhibitory immune signal [36]. In addition to classic monoclonal antibodies, bispecific antibodies are also being developed, targeting both TIGIT and other immune checkpoints, in particular PD-1 or PD-L1, which allows for a synergistic effect [37].

The most advanced therapeutic strategies are based on monoclonal antibodies directed against TIGIT. Tiragolumab is the most promising antibody in this group. In 2021, it received breakthrough therapy designation by the FDA for the treatment of NSCLC with high PD-L1 expression [38]. Other examples include IgG1 antibodies, such as vibostolimab, which enhances T-cell activity by counteracting inhibitory signals in the tumor microenvironment [39]. Etigilimab, another anti-TIGIT antibody, is currently being studied both as monotherapy and in combination with other checkpoint inhibitors, allowing for the simultaneous modulation of different immune pathways [40].

**Figure 4:**
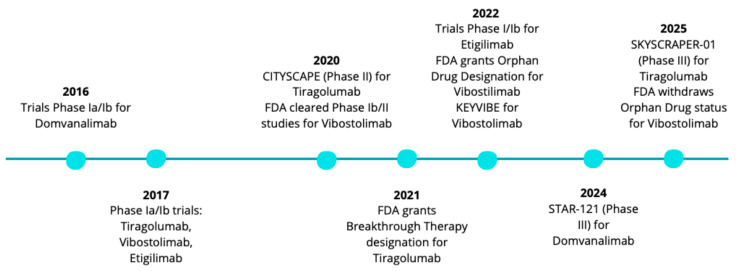
Timeline of clinical development of anti-TIGIT antibodies [38,39,40,41].

In addition to classic monoclonal antibodies, innovative therapeutic approaches are also being developed. Bispecific antibodies combine TIGIT blockade with PD-1 or PD-L1 receptors, potentially enhancing the immunostimulatory effect. Experimental studies also include cell therapies in which T lymphocytes or NK cells are modified to silence TIGIT expression, leading to increased antitumor activity [41]. Figure 4 presents a timeline summarizing clinical studies on TIGIT inhibitors.

TIGIT blockade strategies therefore include both classic monoclonal antibodies and innovative bispecific and cellular approaches, allowing for a broad spectrum of immunological interventions in NSCLC. Current preclinical and early-phase studies indicate that these therapies have the potential to significantly enhance the immune response and become an important component of combination therapy in the future.

Table 1 provides an overview of the main anti-TIGIT antibodies currently investigated in NSCLC, including their immunoglobulin subclass, clinical development stage, combination partners, and regulatory status.

## 6. Clinical Use of Anti-TIGIT in Non-Small-Cell Lung Cancer

Clinical trials of TIGIT inhibitors, such as vibostolimab and tiragolumab, focus on assessing safety, tolerability, and efficacy in the treatment of NSCLC.

A phase I study evaluated the safety, tolerability, and efficacy of vibostolimab as monotherapy or in combination with pembrolizumab. Patients were divided into two cohorts. Group A consisted of 76 patients with advanced solid tumors (34 monotherapy, 42 combination therapy) and group B consisted of 106 patients with NSCLC. Patients in group B were divided according to their resistance to anti-PD-1/PD-L1 therapies. The first group (I) consisted of 67 resistant patients (34 monotherapy, 33 combination therapy), and the second group (II) consisted of 39 patients who had not previously used anti-PD-1/PD-L1 and all received combination therapy. The results showed that vibostolimab achieved an acceptable safety and tolerability profile. The most commonly observed TRAEs were pruritus, hypoalbuminemia, fever, pain, lymphopenia, and rash. Grade 3–5 adverse events occurred in approximately 15% of patients treated for NSCLC. The incidence of individual TRAEs is presented in Table 2 [42].

Another study evaluated the safety and efficacy of tiragolumab in the treatment of patients with advanced solid tumors, including NSCLC. Phase 1a evaluated the safety of tiragolumab administered as monotherapy, and phase 1b evaluated its safety in combination with atezolizumab. Twenty-four patients with NSCLC participated in phase 1a and 49 in phase 1b. No dose-limiting toxicity was observed in either phase. The recommended Phase 2 dose was 600 mg of tiragolumab administered every 3 weeks. The most commonly observed TRAEs included fatigue, pruritus, and joint pain. Grade 3–5 adverse events occurred in approximately 4% of patients in both phases of the study. The incidence of immune-related adverse events was higher in phase 1b (59%) compared to phase 1a (17%), with the most common symptoms being rash and hepatitis. In addition, the efficacy of the therapy was evaluated. In the NSCLC patient cohort (n = 13), the confirmed ORR was 46%, and the median DOR was not reached. In the cohort of patients with esophageal cancer (n = 18), the ORR was 28%, and the median DOR was 15.2 months (95% CI: 7.0–not reached). It is worth noting that these patients had not previously been treated with immunotherapy [43].

The randomized, double-blind phase II CITYSCAPE study evaluated the efficacy of tiragolumab in combination with atezolizumab as first-line therapy in patients with NSCLC. The study included 135 patients who were randomly assigned to two groups: the study group (tiragolumab + atezolizumab, n = 67) and the control group (placebo + atezolizumab, n = 68). The drugs were administered intravenously every three weeks at doses of 600 mg tiragolumab and 1200 mg atezolizumab, respectively. The results showed that the addition of anti-TIGIT to anti-PD-L1 therapy significantly improved ORR. In the tiragolumab and atezolizumab group, ORR was 31.3% (21/67; 95% CI: 19.5–43.2; *p* = 0.031), while in the placebo plus atezolizumab group it was 16.2% (11/68; 95% CI: 6.7–25.7). The median PFS was 5.4 months in the study group and 3.6 months in the control group, with a hazard ratio (HR) of 0.57 (95% CI: 0.37–0.90; *p* = 0.015). The CITYSCAPE study provided the first evidence of the clinical activity of TIGIT blockade in combination with a PD-L1 inhibitor in patients with previously untreated, PD-L1-positive NSCLC. The combination of tiragolumab and atezolizumab improved ORR and PFS compared to atezolizumab alone, while maintaining an acceptable safety profile. However, it should be emphasized that this was a phase II trial with a limited number of patients, and the results needed to be confirmed in phase III trials, which unfortunately did not replicate the observed advantage [44].

However, it should be emphasized that although the results of phase II were promising, the subsequent phase III SKYSCRAPER-01 study did not confirm the superiority of tiragolumab in combination with atezolizumab, indicating the need for further research in selected patient populations. The study involved 534 patients who were divided into two groups: a control group receiving tiragolumab+atezolizumab and a placebo+atezolizumab control group. Analysis of the primary endpoints, including overall survival (OS) and PFS, did not show a statistically significant advantage of the experimental therapy in the entire population. The median PFS in the study group was 7.0 months (95% CI: 5.6–9.8) compared to 5.6 months (95% CI: 4.4–7.0) in the control group. Similarly, OS was 23.1 and 16.9 months, respectively. In addition, ORR and DOR were similar in both groups, suggesting no significant positive effect of tiragolumab on these parameters [45].

A retrospective study evaluated the impact of concurrent chemoradiotherapy (cCRT) on the expression of immune checkpoints in tumor tissues of patients with NSCLC (n = 55). It was shown that in patients undergoing cCRT, compared to systemic treatment, an increase in PD-L1 expression was significantly more frequent (50.0% vs. 5.0%; *p* < 0.001) and TIGIT (41.5% vs. 34.0%; *p* = 0.008) compared to systemic therapy, while differences in PD-1 and CD155 expression did not reach statistical significance. Importantly, high concurrent expression of PD-L1 and TIGIT after cCRT was associated with shorter OS (*p* = 0.008), while single expression of either marker was not clearly prognostic. These results suggest that cCRT may induce immunosuppression in the tumor microenvironment by enhancing PD-L1 and TIGIT pathways. This highlights the potential value of therapies that block both checkpoints simultaneously, especially in patients undergoing chemoradiotherapy [46].

Currently, only a few studies evaluating the efficacy of TIGIT blockade in NSCLC therapy have been completed, including phase I and II trials, which provided the first evidence of the clinical activity of this strategy. In summary, TIGIT inhibitors show promising results in early-phase studies, especially in combination with PD-1/PD-L1 inhibitors. However, the lack of confirmation of these benefits in phase III studies highlights the need for further analysis and identification of predictive biomarkers. Several large, randomized Phase II and III trials are still ongoing, and their results will be crucial in determining the place of anti-TIGIT therapy in clinical practice. One such trial is the Phase III STAR121 trial, involving 720 patients with NSCLC [47]. In addition, according to the ClinicalTrials.gov database, there are currently 17 clinical trials registered to evaluate the efficacy and safety of anti-TIGIT antibodies in patients with NSCLC. Although time is needed to expand the pool of available data, it should be emphasized that ongoing trials may represent a breakthrough in the systemic treatment of NSCLC and potentially other solid tumors in the future.

Table 3 provides a summary of the studies included in this systematic review, highlighting their key characteristics, patient populations, interventions, and main findings.

## 7. Discussion

Research into TIGIT as a therapeutic target for NSCLC reflects a growing awareness that resistance to PD-1/PD-L1 blockade remains a key limitation of current immunotherapy. Preclinical and translational data clearly show that TIGIT is co-expressed with PD-1 on exhausted CD8+ T lymphocytes and NK cells, contributing to the weakening of their effector functions and the escape of the tumor from immune surveillance. Importantly, simultaneous blockade of TIGIT and PD-1 leads to synergistic restoration of cytotoxic activity, providing a strong biological rationale for clinical trials.

However, the results of clinical trials are more complex. While early studies (phase I–II) using antibodies such as tiragolumab and vibostolimab showed a favorable safety profile and initial signs of efficacy, especially in combination therapy with PD-L1 inhibitors, key phase III studies, including the SKYSCRAPER -01, did not confirm the previously observed benefits [42,43,47].

A critical aspect in interpreting these findings is the discrepancy between the phase II CITYSCAPE and the phase III SKYSCRAPER-01 trial. The encouraging benefit observed in CITYSCAPE may partly reflect the smaller sample size, exploratory endpoints, and the enrichment for patients with higher PD-L1 expression (≥1%). In contrast, SKYSCRAPER-01 enrolled a much larger and more heterogeneous population of patients with high PD-L1 expression (≥50%), and the primary endpoints were powered for overall survival and progression-free survival rather than exploratory efficacy signals.

Differences in trial design, statistical power, and patient selection—including potential variations in tumor mutational burden, smoking status, and co-expression of CD226—could explain why the phase II signal was not reproduced in phase III. This suggests that TIGIT blockade may only benefit specific biomarker-defined subgroups, and highlights the need for future biomarker-driven clinical strategies.

Another important consideration is that TIGIT blockade may not be universally effective but rather limited to biomarker-defined subgroups. Translational studies indicate that TIGIT expression alone is insufficient to predict response; the functional balance with CD226 and the presence of its ligands, particularly CD155, may be decisive.

In the context of biomarkers, measuring TIGIT or PD-L1 expression alone proves insufficient to predict treatment response. Increasing attention is being paid to the balance between inhibitory (TIGIT) and costimulatory (CD226) signals. Low CD226 levels on CD8^+^ lymphocytes have been shown to be associated with their dysfunction and poorer response to immunotherapy, making this marker a potential predictive biomarker [23]. Furthermore, modern analytical techniques—such as single-cell RNA sequencing—allow for a better understanding of the heterogeneity of T-cell subpopulations in the tumor microenvironment, while spatial transcriptomics enables mapping of checkpoint expression within the context of tissue architecture. Integrating these technologies may, in the future, enable the development of complex biomarker panels that will more precisely identify patients who will benefit from TIGIT blockade.

The discrepancy between CITYSCAPE and SKYSCRAPER-01 highlights the limitations of relying solely on PD-L1 expression for patient selection. Moreover, chemoradiotherapy has been shown to increase TIGIT and PD-L1 levels, suggesting that timing and treatment context may critically influence outcomes. Advanced technologies such as single-cell RNA sequencing and spatial transcriptomics may enable identification of responsive patient subsets and reveal mechanisms of resistance, including loss of CD226 or compensatory upregulation of alternative checkpoints such as LAG-3 or TIM-3.

Thus, the apparent failure of SKYSCRAPER-01 should not be interpreted as the end of TIGIT-targeted strategies, but as a call to refine patient selection and explore rational combinatorial approaches. This discrepancy highlights two fundamental issues: the need to identify reliable predictive biomarkers and the need for appropriate patient stratification. It is becoming increasingly clear that the efficacy of TIGIT blockade will not be uniform across all NSCLC subgroups. PD-L1 expression levels, CD226 co-expression, or the presence of specific molecular subtypes (e.g., KRAS mutations) may determine the response to therapy.

Although TIGIT and its ligand CD155 may show variable expression between histological subtypes of NSCLC (adenocarcinoma vs. squamous-cell carcinoma), currently there is insufficient clinical evidence to recommend differential use of anti-TIGIT therapy based on histology alone. Thus, histological subtype is not used as a primary criterion for patient selection, although further studies may clarify potential subtype-specific effects.

It is also worth emphasizing the dynamic role of the tumor microenvironment. Increased expression of PVR/CD155 after chemoradiotherapy or PD-L1 blockade indicates adaptive mechanisms of tumor resistance. This suggests that TIGIT inhibitors may be most effective in combination regimens, especially in patients previously treated with anti-PD-1/PD-L1 or after cCRT. Bispecific antibodies (TIGIT + PD-1/PD-L1) and cell therapies with silenced TIGIT expression in T or NK lymphocytes are also promising avenues for development [46].

The failure of some phase III studies should not be seen as proof of the concept’s ineffectiveness, but rather as a signal to improve clinical strategies. A similar situation occurred in the early stages of anti-PD-1/PD-L1 therapy development, where only a precise biomarker-based approach enabled its breakthrough application. In this context, the integration of TIGIT, CD155, and PD-L1 expression into predictive panels may significantly improve patient selection.

Finally, TIGIT should be considered not only as a therapeutic target, but also as a prognostic and predictive biomarker. Numerous studies indicate that high expression of TIGIT/CD155 is associated with a poorer prognosis, which further enhances its clinical potential.

An important aspect that requires further development is spatial and single-cell technologies that allow for a more precise assessment of the co-occurrence of TIGIT expression with other checkpoints within the tumor microenvironment. These technologies will make it possible to better predict which patient groups can realistically benefit from TIGIT blockade [48].

New immune checkpoints are being explored as complementary targets. LAG-3 and T-cell immunoglobulin and mucin domain-containing protein 3 (TIM-3) act as inhibitory receptors that contribute to T-cell exhaustion through mechanisms such as binding to Major Histocompatibility Complex (MHC) class II and interactions with galectin-9, while Tumor necrosis factor receptor superfamily member 4 (OX40) serves as a costimulatory receptor that enhances T-cell activation and proliferation. Combining TIGIT blockade with these approaches, particularly simultaneous inhibition of multiple checkpoints or pairing inhibitory and activating pathways, is being investigated in preclinical and early-phase studies. Such strategies may help overcome resistance and improve the overall efficacy of immunotherapy in NSCLC [15,41].

In contrast to previous reviews on TIGIT in NSCLC, our systematic review is distinguished by its unique contribution, focusing on the most recent evidence (2022–2025) and by integrating mechanistic insights into the TIGIT/CD226 axis with clinical trial outcomes. We specifically analyze the discrepancies between early-phase (CITYSCAPE) and late-phase (SKYSCRAPER-01) results, and highlight novel therapeutic strategies beyond PD-1/PD-L1 blockade, such as bispecific antibodies and TIGIT-targeted cellular therapies. Importantly, we emphasize the urgent need for biomarker-driven approaches—particularly the combined assessment of PD-L1, CD155, and CD226—to optimize patient selection. Taken together, this comprehensive and up-to-date perspective provides added value compared with earlier publications and outlines directions for future translational and clinical research.

Our systematic review highlights three major findings. First, TIGIT blockade shows synergistic effects when combined with PD-1/PD-L1 inhibitors, although efficacy is inconsistent across trial phases. Second, the TIGIT/CD226/PVR axis emerges as a crucial determinant of therapeutic response, underscoring the importance of biomarker-driven patient selection. Third, novel approaches such as bispecific antibodies and TIGIT-targeted cell therapies represent promising avenues for overcoming resistance. Collectively, these insights emphasize that the future success of anti-TIGIT strategies in NSCLC will depend on precise biomarker integration and rational combination therapies.

## 8. Conclusions

Anti-TIGIT therapies represent a highly promising immuno-oncology strategy for NSCLC. TIGIT blockade can enhance antitumor immunity by restoring T-cell and NK cell function, potentiating responses that are often suppressed within the tumor microenvironment. Early clinical studies suggest benefit, particularly in combination with PD-1/PD-L1 inhibitors; however, current evidence is limited by small sample sizes, heterogeneous patient populations, and short follow-up periods.

Future research should focus on identifying predictive biomarkers for precise patient selection, optimizing rational combination strategies, and developing novel modalities such as bispecific antibodies and engineered cell therapies. Well-designed, randomized trials with stratified cohorts will be essential to confirm these observations. If validated in phase III studies, TIGIT blockade could become an integral part of standard NSCLC therapy.

## Figures and Tables

**Table 1:** Overview of anti-TIGIT antibodies in clinical development for NSCLC [38,39,40,41].

Antibody	IgG Subclass	Clinical Phase	Combination Partner	FDA Status
Tiragolumab	IgG1, Fc-competent	II-III	Atezolizumab (PD-L1)	Breakthrough Therapy Designation
Vibostolimab	IgG1, Fc-competent	Ib-II	Pembrolizumab (PD-1)	Orphan Drug Designation (SCLC) withdrawn
Etigilimab	IgG1, Fc-engineered	I-Ib	Nivolumab (PD-1)	FDA IND clearance (early stage)
Domvanalimab	IgG1, Fc-competent	III	Zimberelimab (PD-1)	No FDA approval

**Table 2:** Frequency of TRAEs of vibostolimab in patients with NSCLC [42].

Resistance to PD-1/PDL-1	No Resistance	Resistance	
**Type of therapy:**	Combination therapy	Monotherapy	Combination therapy
Itching	38%	9%	36%
Hypoalbuminemia	31%	3%	0%
Fever	21%	6%	3%
Lymphopenia	18%	0%	0%
Rash	15%	21%	21%
Fatigue	13%	21%	24%
Joint pain	5%	12%	0%
Reduced appetite	5%	9%	12%
Nausea	3%	12%	6%

**Table 3:** Summary of Studies Included in the Systematic Review.

Year	Phase	Population	PD-L1 Requirement	Intervention	Results	Citation
2022	I	182	-	Vibostolimab	Safety assessed; most common TRAEs: pruritus, hypoalbuminemia, fever, pain, lymphopenia, rash	[42]
2023	Ia/Ib	24/49	-	Tiragolumab	ORR 46% DOR not reached	[43]
2022	II	135	PD-L1 ≥ 1%	Tiragolumab	ORR 31.3% Median PFS 5.4 month	[44]
2025	III	534	PD-L1≥ 50%	Tiragolumab	Median PFS 7.0 month OS 23.1 month	[45]
2023	Retrospective	55	-	cCRT	Elevated PD-L1 and TIGIT expression post-cCRT associated	[46]
2024	III	720	-	Domvanalimab	Ongoing	[47]

## Data Availability

No new data were created or analyzed in this study. Data sharing is not applicable to this article.

## Abbreviations

The following abbreviations are used in this manuscript:

ALK	Anaplastic Lymphoma Kinase
BRAF	B-Raf Proto-Oncogene
Cbl-b	Ubiquitin–Protein Ligase
cCRT	Concurrent Chemoradiotherapy
CTLA-4	Cytotoxic T-Lymphocyte–Associated Protein 4
DOR	Duration of Response
EOMES	Eomesodermin
ERK 1/2	Extracellular Signal-Regulated Kinases 1 and 2
FDA	Food and Drug Administration
FOXO1	Forkhead Box O1
FOXP3	Forkhead Box P3
Grb2	Growth Factor Receptor-Bound Protein 2
HER2	Human Epidermal Growth Factor Receptor 2
HR	Hazard Ratio
IFN-γ	Interferon Gamma
ICI	Immune Checkpoint Inhibitor
ITIM	Immunoreceptor Tyrosine-Based Inhibitory Motif
KRAS	Kirsten Rat Sarcoma Viral Oncogene Homolog
LAG-3	Lymphocyte Activation Gene 3
LC	Lung Cancer
MAPK	Mitogen-Activated Protein Kinase
MET	Mesenchymal–Epithelial Transition Factor
MHC	Major Histocompatibility Complex
NK	Natural Killer
NKT	Natural Killer T
NSCLC	Non-Small-Cell Lung Cancer
ORR	Objective Response Rate
OX	Tumor necrosis factor receptor superfamily member 4
PD-1	Programmed Cell Death Protein 1
PD-L1	Programmed Death-Ligand 1
PFS	Progression-Free Survival
PVR	Poliovirus Receptor
RCT	Randomized Controlled Trials
RET	REarranged during Transfection
ROS1	C-Ros Oncogene 1
SCLC	Small-Cell-Lung Cancer
SHIP-1–Src	Homology 2 Domain-Containing Inositol 5-Phosphatase 1
SH-2–Src	Homology 2
T-BET	T-box Expressed in T Cells
TCE7	Transcription factor 7
T EFF	T Effector Cells
T EM	Effector Memory T Cells
TILs	Tumor-Infiltrating Lymphocytes
TIGIT	T-cell Immunoreceptor with Ig and ITIM Domains
TIM-3	T-cell Immunoglobulin and Mucin Domain-Containing Protein 3
T RM	T tissue-resident memory cells
TRAEs	Treatment-Related Adverse Events
TP53	Tumor Protein p53
ZAP70/Syk	Zeta-Chain-Associated Protein Kinase 70/Spleen Tyrosine Kinase
HPV	Human Papillomavirus
EGFR	Epidermal Growth Factor Receptor
